# Transcriptome analysis of *Lr19*-virulent mutants provides clues for the *AvrLr19* of *Puccinia triticina*

**DOI:** 10.3389/fmicb.2023.1062548

**Published:** 2023-03-22

**Authors:** Zhongchi Cui, Wenyue Wu, Fan Fan, Fei Wang, Daqun Liu, Dianping Di, Haiyan Wang

**Affiliations:** ^1^College of Plant Protection, Technological Innovation Center for Biological Control of Crop Diseases and Insect Pests of Hebei Province, Hebei Agricultural University, Baoding, Hebei, China; ^2^Plant Protection Institute, Hebei Academy of Agricultural and Forestry Sciences, Baoding, Hebei, China

**Keywords:** wheat, *Puccinia triticina*, single nucleotide polymorphism, sequence insertion or deletion, *AvrLr19* candidates

## Abstract

**Introduction:**

Wheat leaf rust caused by *Puccinia triticina* (*Pt*) remains one of the most destructive diseases of common wheat worldwide. Understanding the pathogenicity mechanisms of *Pt* is important to control wheat leaf rust.

**Methods:**

The urediniospores of *Pt* race PHNT (wheat leaf rust resistance gene *Lr19*-avirulent isolate) were mutagenized with ethyl methanesulfonate (EMS), and two *Lr19*-virulent mutants named M1 and M2 were isolated. RNA sequencing was performed on samples collected from wheat cultivars Chinese Spring and TcLr19 infected with wild-type (WT) PHNT, M1, and M2 isolates at 14 days post-inoculation (dpi), respectively. Screening *AvrLr19* candidates by quantitative reverse transcription PCR (qPCR) and Agrobacterium-mediated transient assays in *Nicotiana benthamiana*.

**Results:**

560 genes with single nucleotide polymorphisms (SNPs) and insertions or deletions (Indels) from non-differentially expressed genes were identified. Among them, 10 secreted proteins were screened based on their fragments per kilobase of exon model per million mapped reads (FPKM) values in the database. qPCR results showed that the expression profiles of 7 secreted proteins including PTTG_27471, PTTG_12441, PTTG_28324, PTTG_26499, PTTG_06910, PTTG_26516, and PTTG_03570 among 10 secreted proteins in mutants were significantly different with that in wild-type isolate after infection wheat TcLr19 and might be related to the recognition between *Lr19* and *AvrLr19*. In addition, a total of 216 differentially expressed genes (DEGs) were obtained from three different sample comparisons including M1-vs-WT, M2-vs-WT, and M1-vs-M2. Among 216 DEGs, 15 were predicted to be secreted proteins. One secreted protein named PTTG_04779 could inhibit programmed progress of cell death (PCD) induced by apoptosis-controlling genes B-cell lymphoma-2 associated X protein (BAX) on *Nicotiana benthamiana*, indicating that it might play a virulence function in plant. Taken together, total 8 secreted proteins, PTTG_04779, PTTG_27471, PTTG_12441, PTTG_28324, PTTG_26499, PTTG_06910, PTTG_26516, PTTG_03570 are identified as *AvrLr19* candidates.

**Discussion:**

Our results showed that a large number of genes participate in the interaction between *Pt* and TcLr19, which will provide valuable resources for the identification of *AvrLr19* candidates and pathogenesis-related genes.

## 1. Introduction

Wheat leaf rust caused by *Puccinia triticina* (*Pt*) is one of the most common and severe diseases in the wheat-growing regions worldwide. The yield losses of wheat infected with *Pt* ranges from 5–15% and the yield of wheat infected with leaf rust at the seedling stage can be reduced by 50% or even more (Bolton et al., 2008; Prasad et al., 2020). Considering the impacts of global climate change, temperature and humidity conditions may be more suitable for the proliferation and epidemic of *Pt* in the future, which will further deleteriously impact wheat yields. Control of leaf rust mainly relies on the fungicide application and deployment of cultivars carrying resistance genes. Genetic resistance is the most effective, environmentally safe, and economically feasible approach to reduce the damage caused by *Pt*. However, monoculture of select resistant varieties leads to the host selection pressures that drive *Pt* evolution and promote the continuous emergence of new virulent races, which often leads to the decline of wheat resistant varieties after several years of planting.

To date, over 100 genes conferring leaf rust resistance derived from wheat (*Triticum*) cultivars have been identified and designated as *Lr1* to *Lr79* (Qureshi et al., 2018). Among them, *Lr9*, *Lr19*, *Lr24*, *Lr34*, *Lr37*, *Lr38*, *Lr46*, *Lr47*, *Lr51*, *Lr53*, *Lr68* and *Lr42* are known to confer effective resistance to leaf rust according to the field survey in Sichuan province, China (Gao et al., 2019). *Lr19* was derived from the grass *Agropyron elongatum* and was transferred to the long arm of wheat chromosome 7D (Pourkhorshid et al., 2022). Although resistance of *Lr19* has been overcome by some virulent *Pt* races in Mexico (Huerta and Singh, 1994), Russia (Lind and Gultyaeva, 2007), Ukraine (Elyasi-Gomari and Panteleev, 2006) and India (Bhardwaj et al., 2005), it still showed high resistance to all the collected *Pt* races in China (Zhang et al., 2020). It has been reported that *Lr19* holds the potential to be deployed in combination with other *Lr* genes in the field to confer durable resistance against leaf rust (Mccallum et al., 2021). It has great potential in wheat breeding program in China. Although *Lr19* has not been cloned, yet many molecular markers closely linked to the *Lr19* gene have been developed, such as *SCS265*, *SCS253*, *GB*, *Xwmc221*, *XustSSR2001-7DL*, *Xgwm37* and *Xgwm44*, and two of them (*GB* and *Xwmc221*) proved to be specific to *Lr19* (Kiel et al., 2020). To prevent *Lr19* from being out-competed by newly emergent virulent races and enable the long-term deployment of *Lr19* in leaf rust-resistant wheat varieties, it is key to clone avirulent gene *AvrLr19* that can be recognized by wheat leaf rust resistance gene *Lr19*, determine the molecular mechanism of *AvrLr19* and *Lr19* interactions, and monitor the natural *Pt* population changes in response to *Lr19* resistance.

Genetic mutation is the most important avenue for creating new rust races and genotypes. Ethyl methanesulfonate (EMS) is an alkylating chemical mutagen that generates single nucleotide polymorphisms (SNPs) and insertions and deletions (Indels), resulting in amino acid sequence variation, and finally leads to phenotype changes (Anderson et al., 2016). Mutagenesis integrated with genomic sequencing is an efficient way to study the relationships between phenotypic traits and associated genotypes, leading to the identification of fungal effectors or *Avr* genes. For example, Salcedo et al. (2017) obtained stripe rust mutants through EMS-induced mutation and performed genome sequencing to obtain *AvrSr35* candidate genes, and then verified the candidate genes through co-expression of *AvrSr35* and its corresponding resistance gene in tobacco and wheat to trigger cell death. Li et al. (2020) screened 30 mutant variants from the least virulent isolate generated by EMS mutagenesis and candidates *Avr* genes were determined by sequencing. Transcriptomics has proven to be an instrumental molecular tool to help identify virulence effectors and *Avr* genes (Elmore et al., 2020; Wei et al., 2020; Xu et al., 2020). In the process of the host and pathogen interaction, the underlying transcriptional regulation of gene expression in the plant and pathogen provides clues to their defense and virulence mechanisms, respectively (Rutter et al., 2017).

The gene-for-gene hypothesis proposed by Flor (2003) indicates that only the host with an “*R* gene” is resistant to the corresponding *Avr* gene in pathogens. Jones and Dangl (2006) proposed the famous “ZigZag” model in 2006 to analyze the molecular mechanism of interaction between plants and their pathogens. In order to inhibit plant defense responses, pathogens secrete a series of effector proteins through the haustorium, the structure with which the fungus attaches to and extracts nutrients from its host plant to interfere with or ablate the plant defense response, so as to meet their own growth needs. In recent years, with the continuous improvement of sequencing technology and the reduction of sequencing costs, and the development and application of prediction software such as SignalP (Almagro Armenteros et al., 2019), TargetP (Emanuelsson et al., 2000), TMHMM (Krogh et al., 2001), EffectorP (Sperschneider et al., 2016) and Pfam (Finn et al., 2014) have improved the screening efficiency of candidate *Avr* genes of rust. To date, a handful of *Avr* genes have been identified in rust pathogens, including *AvrL567*, *AvrP123*, *AvrP4*, *AvrM*, *AvrL2* and *AvrM14* from the flax rust pathogen (Anderson et al., 2016; Prasad et al., 2019), as well as *RTP1* in bean rust (Kemen et al., 2005), and *PGTAUSPE10–1* from wheat stem rust (Petre et al., 2014). Three articles reported the successful cloning of the *Avr* genes *AvrSr50* (Chen et al., 2017) and *AvrSr27* (Upadhyaya et al., 2021) of wheat stem rust, and preliminarily revealed their interaction with corresponding resistance genes (Segovia et al., 2016; Bakkeren and Szabo, 2020). Some effector candidates for *Lr26*, *Lr9*, *Lr24* and *Lr20* were obtained from *Pt* (Wu et al., 2017; Figueroa et al., 2020), but their biological functions have not been determined, so, no known *Avr* genes have been identified in *Pt* so far.

In this study, we aim to obtain *Lr19-*virulent mutants using EMS mutagenization, then identify differentially expressed genes (DEGs) and the genes with SNPs and Indel associated with the *Pt* infection. Our results will provide resources for identification of *AvrLr19* candidates and pathogenicity-related genes, and lay a foundation for revealing the pathogenic mechanism of *Pt*.

## 2. Materials and methods

### 2.1. Plant materials and *Pt* isolates

Wheat seedlings of near-isogenic lines were requested from the Cereal Disease Lab of the USDA located at the University of Minnesota and were preserved in the laboratory of Leaf rust, Hebei Agricultural University. *Pt* race 07–10–426-1 (PHNT race), collected from China and preserved in our laboratory, was used to inoculate wheat according to Roelfs (1984) standards, it was incompatible with the resistant variety TcLr19 (origin of grain resource: Tc*6/RL6040, containing wheat leaf rust resistance gene *Lr19*, *Lr19*+), but compatible with the susceptible variety Chinese Spring (*Lr19*-). Seedlings (14 days old) from wheat lines Chinese Spring (*Lr19*-) inoculated by spraying with a suspension of *Pt* urediniospores were used as controls.

### 2.2. Staining and microscopic observation

To identify fungal tissues and the presence of dead cells in the leaf mesophyll of infected plants, the samples were stained *via* the Rohringer (1997) fluorescent staining method. Stained tissues were observed under optical microscopy, and at least 10 fungal infection sites from three biological replicates were analyzed for each stained leaf sample.

### 2.3. EMS mutagenesis of *Pt* urediniospores of the PHNT race

The *Lr19*-avirulent *Pt* race PHNT was used to create *Lr19*-virulent mutants by treatment with EMS as previously described (Salcedo et al., 2017). Four different concentrations of EMS were used for mutagenesis: 0.1 M, 0.05 M, 0.01 M and 0.005 M. After 2 hours (h) of treatment with EMS, then the urediniospores was washed 1 L of ddH_2_O and dried for 12–14 h at room temperature (22–25°C). The germination rate of spores was observed using microscopy and untreated *Pt* spores were used as control. Spores with at least 50% germination rate were applied with a brush to the surface of the primary leaves of differential wheat near-isogenic lines that carry different *Lr* genes to validate that only *Lr19*-virulence was affected by EMS mutagenesis. After inoculation, the plants were kept at 100% relative humidity in the dark for 24 h at 20°C, and then were incubated in a growth chamber at 22°C with a 16 h/8 h (day/night) photoperiod. In addition, the molecular marker of *Lr19* was used to detect the validity of TcLr19 (Gupta et al., 2006).

### 2.4. Transcriptome sequencing and identification of the *AvrLr19* candidates

The *Pt* pathotypes PHNT mutants and PHNT were purified, and then inoculated onto the TcLr19 and Chinese Spring, respectively. We sampled the inoculated leaf at 14 days post-inoculation (dpi) showing severe disease phenotypes for extraction of total RNA of *Pt*. Transcriptome sequencing and analyses were conducted by OE Biotech Co., Ltd. (Shanghai, China). The RNA was isolated using an RNA isolation kit (RNeasy Plant MiniKit, Tiangen, Beijing) according to the manufacturer’s instructions. Sequencing was carried out on the Illumina HiSeqTM 2500 platform. Then, the clean reads were mapped to reference genome “*Pt* 1–1 BBBD Race 1” (Kiran et al., 2016) using Hisat2 (Trapnell et al., 2010) to obtain the location information on the reference genome or gene, as well as the specific sequence feature information of the sequenced sample. The read counts of each gene were obtained by HTSeq-count (Anders et al., 2015). DEGs were identified using the DESeq R package (Wang et al., 2009). “FDR-adjusted value of *p* <0.05” and “|fold Change| >2” were set as the thresholds for significantly differential expression. Hierarchical clustering analysis of DEGs was performed to explore gene expression patterns. GO enrichment and KEGG (Kanehisa et al., 2008) pathway enrichment analysis of DEGs were, respectively, performed using R based on the hypergeometric distribution. We detected the presence of an N-terminal signal peptide through SignalP 5.0. The expression of genes with SNPs and Indels in interaction between *Pt* and wheat were determined. The expression of genes was profiled based on their fragments per kilobase of exon model per million mapped reads (FPKM) values in the transcriptome database (PRJNA605036). Conserved domains analysis was performed using InterProScan^1^ and HMMER^2^. Effector P 3.0^3^ was used to predict the effector candidate.

### 2.5. qPCR analysis

Total RNA was extracted from leaves infected by *Pt* at 0, 12, 48 and 96 hours post inoculation (hpi). First-strand cDNA was synthesized from an equal amount of RNA using a PrimeScript Reverse Transcriptase kit (TaKaRa, Beijing, China). The *β-actin* gene (GenBank accession ADAS02000088.1: 291774-292,960) encoding *β*-actin protein was used as an internal reference gene, and 3 independent biological replicates were used in the experiments. The quantitative reverse transcription PCR (qPCR) reactions were conducted using a TransStart R Top Green qPCR SuperMix (TransGen, Beijing, China) with a Roche-LightCycler 96 qPCR instrument (Roche, Basel, Switzerland) using different primers designed according to different candidates. The transcriptional abundance of genes was quantitated relative to that of the *β-actin* following the 2^−ΔCT^ method (Ridout et al., 2006).

### 2.6. *Agrobacterium*-mediated transient assays in *Nicotiana benthamiana (N. benthamiana)*

To generate constructs for transient expression in *N. benthamiana*, 4 secreted proteins genes were amplified using primers listed in Supplementary Table S6. The resulting PCR products were cloned into the in-house ligation-independent cloning vector pCamA carries a CaMV 35S promoter The resulting pCamA::secreted proteins constructs were used for Agrobacterium transformation, as described previously (Ma et al., 2012).

### 2.7. Yeast secretion trap assay

The predicted coding sequences of the signal peptides of selected effector candidates were amplified from cDNA using the primers listed in Supplementary Table S6 and cloned into the yeast secretion trap vector pSUC2 using *EcoR* I and *Xho* I restriction sites. Ps87 were used as a positive control, Mg87 and YTK12 as negative control. The resulting constructs were transformed into the yeast strain YTK12 to examine secretion following the method described previously (Haddadi et al., 2016). And the reduction of colorless TTC to red-colored insoluble TFP, as described by Yin et al. (2018).

## 3. Results

### 3.1. *Lr19* Triggers a resistance response at the early stages of infection source identification initiative

**Figure 1:**
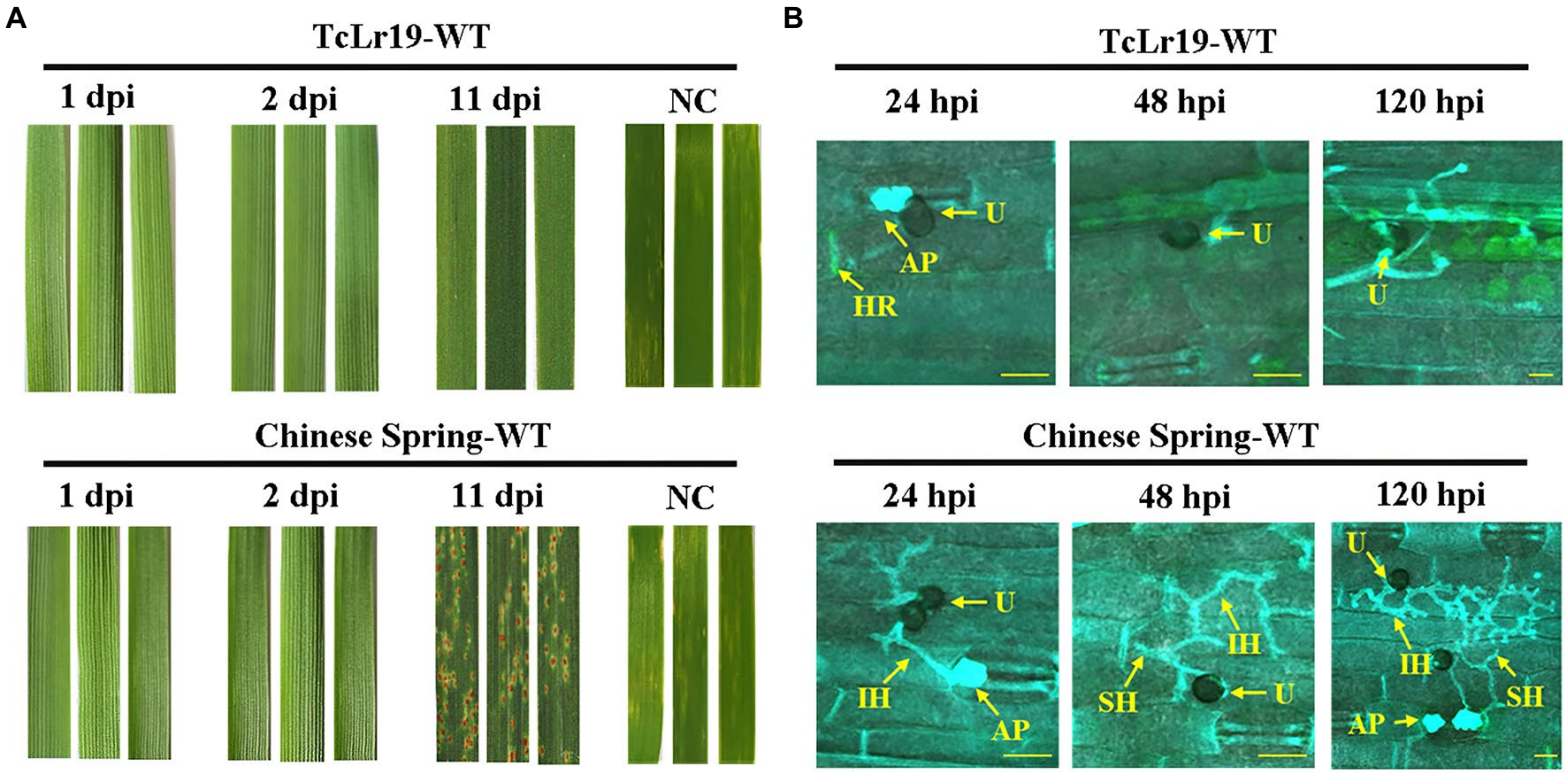
*Lr19* provides prehaustoria resistance against *Pt.* **(A)** Infected leaves of susceptible cultivar Chinese Spring (*Lr19*-) and resistant line TcLr19 (*Lr19*+) were observed; NC: negative control, non-inoculation. **(B)** Fungal infection hyphae (IH) (stained blue) entered the leaf mesophyll tissue (stained blue) through the plant stoma (S) in both wheat lines at 24 hpi. Fungal haustoria (H) developed only in susceptible Chinese Spring. Imaging at 48 hpi revealed fungal growth in susceptible Chinese Spring, but no further fungal growth in TcLr19. At 48 hpi revealed presumably dead host cells (stained green) in TcLr19; no dead cells were revealed in Chinese Spring. U, spores; AP, appressorium. Scale bars, 100 μm.

Leaf rust resistance conferred by *Lr19* is best expressed in all stages of the wheat plant and culminates in a hypersensitive response (HR) at the infection site which is also known as race-specific resistance. In order to confirm the recognition between *Lr19* and its respective *Avr* gene in the *Lr19*-avirulent *Pt* race PHNT, the phenotype and histopathology examination of *Pt*-infected leaf tissues from resistant TcLr19 (*Lr19*+) and susceptible Chinese Spring (*Lr19*–) wheat lines were analyzed. Compared to Chinese Spring (*Lr19*–) wheat lines, the development of fungal infection hyphae (stained blue) stopped before the formation of a haustorium, the structure with which the fungus extracts nutrients from its host plant, in the resistant wheat line TcLr19 (*Lr19*+). Imaging at 48 hpi revealed fungal growth in susceptible Chines Spring but no further fungal growth in TcLr19, the dead host cells in TcLr19 were stained green, and no dead cells were revealed in Chinese Spring (Figures 1A,B). This early immune response is consistent with pronounced HR symptoms and suggested early expression of a fungal gene recognized by *Lr19*, which demonstrated that *Lr19* triggers a resistance response at the early stages of infection.

### 3.2. *Lr19*-virulent mutants were obtained and confirmed

**Figure 2:**
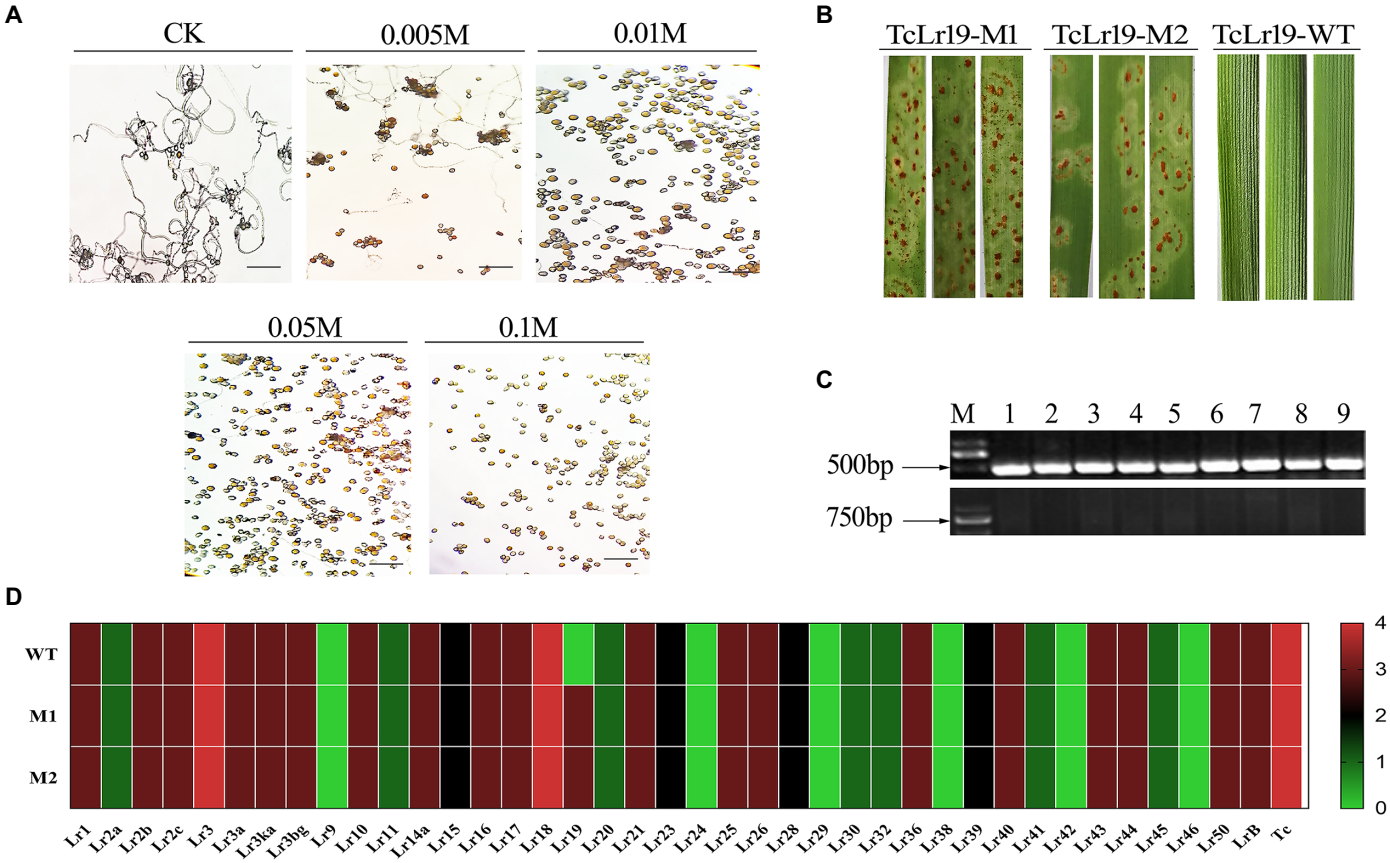
*Lr19*-virulent Mutants were obtained and confirmed. **(A)** The germination effect of *Pt* spores of PHNT race under different EMS concentration; CK, control check. **(B)** The phenotypes of *Lr19*-virulent Mutants M1 and M2 were compared with wild-type (WT). **(C)** Molecular markers detection of *Lr19*. There was a specific band at 500 bp for forward primer detection and there was no specific band at 750 bp for reverse primer detection, M is the abbreviation of marker, 1–9 represents 9 test samples. **(D)** Heatmap of WT and its mutants (M1 and M2) of *Pt* based on infection types, the virulence characterization of all isolates was conducted on the 36 wheat *Lr* single-gene differentials. Its 0 to 4 were transformed to the color key ranging from green to red, which indicate avirulent (resistant) to virulent (susceptible) reactions.

The *Lr19*-avirulent *Pt* race PHNT was used to create *Lr19*-virulent mutants *via* EMS mutagenesis. The germination effect of PHNT urediniospores under different EMS concentrations was observed, and control check (CK) were carried out using 0 M EMS. The results showed that the germination rate of urediniospores was 50% at an EMS concentration of 0.005 M (Figure 2A). Two *Pt* mutants virulent to the *Lr19* allele were isolated, named M1 and M2, suggesting that they contained mutations in the *Avr* factor (Figure 2B). Confocal microscopy of wheat leaves from the compatible Chinese Spring cultivar infected with the wild-type (WT) and mutant *Pt* strains. The length of the hyphae in Chinese Spring inoculated with M1 and M2 was the same as Chinese Spring inoculated with WT. The results showed that the *AvrLr19* gene mutation did not change the virulence of the pathogen (Supplementary Figure S1). In order to ensure the reliability of experimental materials of TcLr19, molecular markers of *Lr19* were used to detect the existence of *Lr19* (Figure 2C). To validate that only *Lr19*-virulence was affected by the EMS mutagenesis, M1 and M2 were analyzed by infecting a standard full set of near-isogenic lines that carry different *Lr* genes, with the WT PHNT race inoculated as controls (Figure 2D; Supplementary Table S1). Only TcLr19 inoculated with M1 and M2 demonstrated a change from resistant (‘0’) to susceptible (‘3 +’), while the susceptibility of other near-isogenic lines remained unchanged, indicating that *AvrLr19* was altered in M1 and M2 alleles and that *Lr19*-virulent mutant strains were successfully obtained.

### 3.3. RNA sequencing (RNA-Seq) analyses display the information of M1, M2, and WT

As there is no virulent strain of *Lr19* in the field, *Pt*-Chinese Spring which was compatible interaction was used as wildtype isolates to compare with *Pt*-TcLr19 mutants. To characterize the genes containing SNPs and Indels between *Pt*-Chinese Spring and *Pt*-TcLr19 mutants during infection, RNA-seq of TcLr19 or Chinese Spring inoculated with M1, M2, and WT were performed. Following inoculation and incubation, leaves displaying infection symptoms were harvested, then the total RNA was extracted from M1, M2, and WT successfully (Supplementary Figure S2A) and was used for RNA-seq cDNA library construction. Quality statistics of sequencing data and the percentages of reads for each sample were mapped to the *Pt* BBBD race reference genome (Supplementary Tables S2, S3). Based on the density plot (Supplementary Figure S2B), box-whisker plot (Supplementary Figure S3A), and the expected number of FPKM expression distribution plot (Supplementary Figure S3B) of gene expression levels. The results suggested that the differences in the distributions were low between the three repeat libraries of each sample, it is clear that the expression level and number of protein-coding genes in each sample is highly similar. Variability among the samples was determined by preparing a principal component analysis (PCA). The WT-sample was located in the third quadrant. The M1-sample was located in the second and fourth quadrants. The M2-sample is located fourth quadrant and the edge of the third quadrant (Supplementary Figure S3C). MT-sample is separated from WT-sample as shown by PCA analysis. PCA displayed a clear distinction between the transcriptomes of mutant and WT strains.

### 3.4. Characterization of genes containing SNPs and indels derived from whole transcriptome

**Figure 3:**
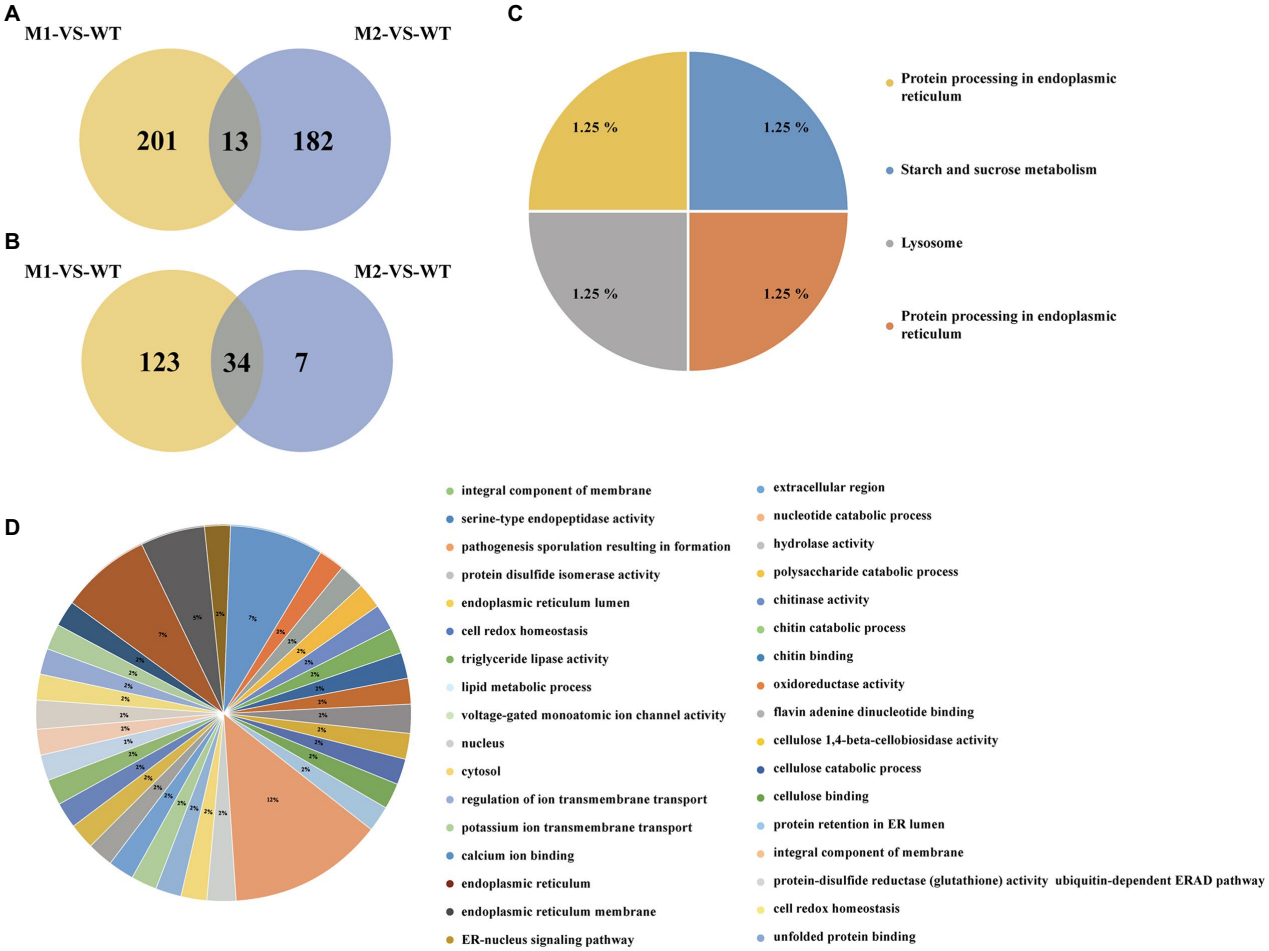
The types and numbers of SNPs in M1, M2, and WT among not differentially expressed genes. **(A)** Numbers of SNPs from the M1-*VS*-WT and M2-*VS*-WT. **(B)** Numbers of Indels from the M1-*VS*-WT and M2-*VS*-WT. **(C)** Results of KEGG analysis of secreted proteins. **(D)** GO annotation for secreted proteins.

EMS mutagenesis generates SNPs and Indels, thereby inducing base substitutions in the genome, and eventually leading to phenotype changes based on mutations. SNPs were screened by selecting only CG to TA mutations since mutations caused by EMS had the strong CG to TA transition bias. Firstly, considering that *AvrLr19* might be non-functional in two mutants and be strongly repressed in expression, alignment and mutational analysis was done in whole transcriptome level except the DEGs. After comparison of sequences and keeping only the EMS-induced SNPs and Indels, a total of 396 genes containing SNPs and 164 genes containing Indels were identified from non-differentially expressed genes (Figures 3A,B), of which 45 genes encoding secreted proteins were predicted to be candidate genes (Table 1). Missense_variants were the predominant (96.67%) among all the deleterious mutations. Similarly, the effects of Indels were mostly in 5’-UTRs (26.67%), 3’-UTRs (26.67%), upstream (20.00%) and frameshift_variant (20.00%) (Supplementary Tables S4, S5). Through the analysis of KEGG enrichment, we explored the main biochemical metabolic pathways and signal transduction pathways involved in secreted proteins. The results show that 4 genes are distributed among “protein processing in endoplasmic reticulum,” “starch and sucrose metabolism,” “lysosome” and “protein processing in endoplasmic reticulum” pathways (Figure 3C). GO analysis was performed to classify the 45 genes into 34 classes categories, 12% genes are enriched in terms of “integral component of membrane” (Figure 3D).

**Table 1:** 45 secreted protein genes contain SNPs and Indels among non-differentially expressed genes.

Gene name	SPL (%)	AA	Polymorphic in *Pt*	Description	Gene	SPL (%)	AA	Polymorphic in *Pt*	Description
*PTTG_03570*	71	115	Yes	–	*PTTG_00455*	64	122	Yes	Secreted protein ARB_01864
*PTTG_00981*	77	245	Yes	–	*PTTG_04011*	97	234	Yes	–
*PTTG_29391*	80	167	Yes	–	*PTTG_26966*	98	324	Yes	–
*PTTG_09239*	52	135	Yes	–	*PTTG_28035*	87	411	Yes	ER-retained PMA1-suppressing protein 1
*PTTG_00016*	88	333	Yes	Alkaline protease 2	*PTTG_02175*	85	111	Yes	Dehydrogenase patE
*PTTG_01827*	72	256	Yes	Protein disulfide-isomerase	*PTTG_05834*	98	167	Yes	–
*PTTG_03567*	67	433	Yes	Lipase 4	*PTTG_28654*	99	187	Yes	–
*PTTG_06895*	55	431	Yes	Putative voltage-gated potassium channel subunit beta	*PTTG_08941*	85	356	Yes	–
*PTTG_27264*	82	378	Yes	–	*PTTG_28961*	81	278	Yes	–
*PTTG_08198*	90	233	Yes	–	*PTTG_26282*	89	321	Yes	–
*PTTG_04053*	92	267	Yes	–	*PTTG_07374*	90	169	Yes	–
*PTTG_02477*	95	198	Yes	–	*PTTG_00363*	92	344	Yes	Kelch repeat-containing protein ARB_01230
*PTTG_28296*	66	166	Yes	UPF0674 endoplasmic reticulum membrane protein C2G5.01	*PTTG_07626*	95	277	Yes	–
*PTTG_25160*	70	277	Yes		*PTTG_26516*	50	143	Yes	–
*PTTG_06256*	69	133	Yes	Probable 1,4-beta-D-glucan cellobiohydrolase B	*PTTG_26586*	98	124	Yes	–
*PTTG_26499*	70	326	Yes	–	*PTTG_11,671*	67	255	Yes	–
*PTTG_27,005*	92	311	Yes	–	*PTTG_12441*	76	134	Yes	–
*PTTG_11739*	88	289	Yes	–	*PTTG_28601*	64	178	Yes	–
*PTTG_27471*	66	177	Yes	–	*PTTG_30601*	78	281	Yes	–
*PTTG_03597*	62	322	Yes	–	*PTTG_06229*	97	176	Yes	–
*PTTG_28324*	59	118	Yes	–	*PTTG_02548*	96	277	Yes	–
*PTTG_06910*	60	343	Yes	–					

SPL, signal peptide likelihood; AA, Amino acid; Polymorphic in *Pt*, The polymorphisms of candidates were determined by searching homologous sequences from publicly available protein database in *Pt*.

### 3.5. Expression profiles of genes containing SNPs and indels in MT-*VS*-WT

**Figure 4:**
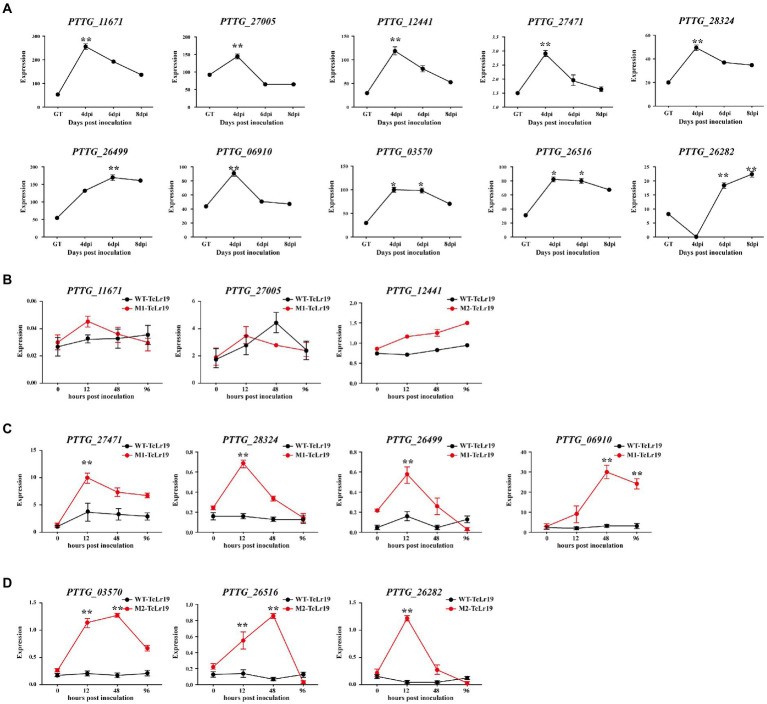
The genes containing SNPs and Indels with different expression patterns were selected. **(A)** Their expression patterns were generated based on corresponding FPKM values from the transcriptome database. The transcript levels of the genes containing SNPs and Indels selected during *Pt* infection at 4, 6, and 8 days post-inoculation (dpi). **(B–D)** Transcriptional profile of genes during the *Pt* pathotypes infection measured by qPCR. The transcript levels of the 14 secreted proteins during *Pt* infection at 0, 12, 48, and 96 hpi (Hour post inoculation) were determined by qPCR assay. The transcript levels for all genes were expressed as linearized fold-*Pt actin* levels, which were calculated according to the formula 2^−△CT^. Data were expressed as mean values ± SE from four biological replicates. An asterisk (*p < 0.05,**p < 0.01) indicates.

In order to confirm whether 45 secreted proteins containing SNPs and Indels are related to the infection of *Pt*, their expression in the interaction between *Pt* race PHNT and wheat was identified based on their FPKM values in a transcriptome database (PRJNA605036). Among them, 9 genes including *PTTG_27471*, *PTTG_27,005*, *PTTG_28324*, *PTTG_06910*, *PTTG_26499*, *PTTG_11671*, *PTTG_26282*, *PTTG_12441*, and *PTTG_26516* with SNPs were significantly upregulated during the early stage (4–6 dpi) of *Pt* infection, and one gene *PTTG_03570* containing Indels were significantly upregulated during 4–6 dpi of *Pt* infection (Figure 4A). All the 10 genes highly expressed at 4–6 dpi deserve more attention than those highly expressed at GT stage.

qPCR was conducted in order to test whether the expression of these 10 genes were affected by EMS mutation. The results displayed that the expression of *PTTG-11671* and *PTTG-27005* in M1-TcLr19 displayed similar profiling to that in WT-TcLr19, and the expression of *PTTG_12441* in M2-TcLr19 also displayed similar profiling to that in WT-TcLr19 (Figure 4B), which indicated that *PTTG-11671*, *PTTG-27005* and *PTTG_12441* expression was not affected by EMS mutation. However, the expression profiling’s of the other 7 genes including *PTTG_27471*, *PTTG_03570*, *PTTG_26282*, *PTTG_28324*, *PTTG_06910*, *PTTG_26516*, and *PTTG_26499* in MT-TcLr19 were significantly different with that in WT-TcLr19 at 12–48 hpi. The expression level of *PTTG_27471*, *PTTG_28324* and *PTTG_26499* in M1 was about 4-fold higher than that in WT at 12 hpi, and *PTTG_06910* expression peaked at 48 hpi in M1 when the expression level was 30-fold higher than that in WT (Figure 4C). In M2, *PTTG_03570* and *PTTG_26516* expression peaked at 48 hpi, and the expression level of *PTTG_03570* and *PTTG_26516* in M2 was about 5-fold higher than that in WT. The transcript quantity of *PTTG_26282* in M2 peaked at 12 hpi which was almost 15-fold higher compared with that in WT (Figure 4D). Taken together, EMS mutation affected the expression of these 7 genes in *Lr19*-virulent mutants, these 7 genes should be further study as *AvrLr19* candidate genes.

### 3.6. Characterization of *Pt* secreted proteins in DEGs

**Figure 5:**
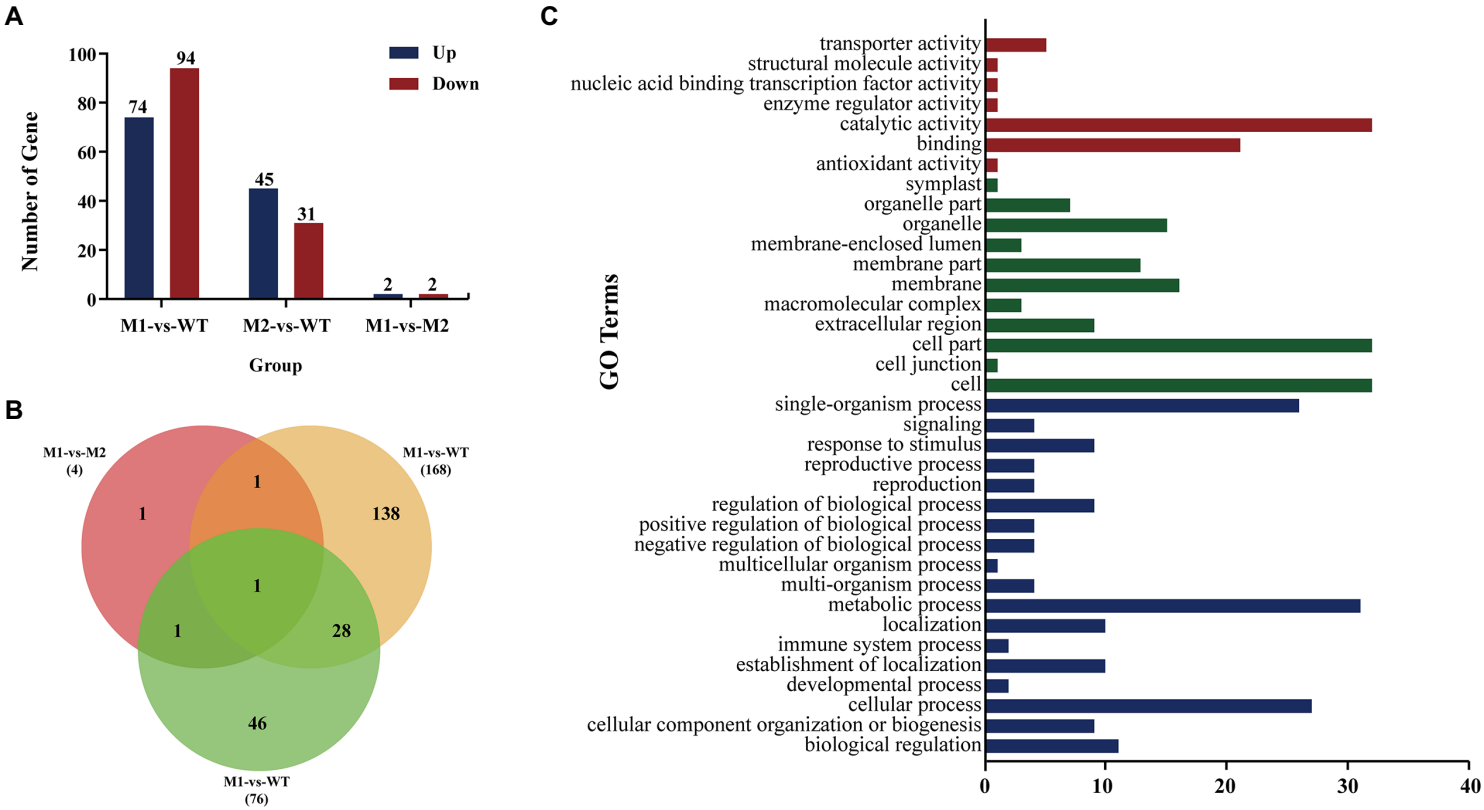
The differentially expressed genes (DEGs). **(A)** Number of up-regulated and down regulated DEGs indifferent samples. **(B)** DEGs that are unique or shared among various samples comparisons in mutants (M1, M2) and WT. The numbers of DEGs are noted in each section of the Venn diagrams. **(C)** Bar graph showing the number of 216 DEGs identified utilizing gene ontology (GO) enrichment analysis for involvement in specific molecular function, cellular component and biological processes. In the figure, red color represents molecular function, green color represents molecular function, blue color represents molecular function, the Y coordinate is the name of GO term, and the X coordinate is number of gene.

The function of *Avr* genes might be affected by changes in expression rather than sequence changes (Upadhyaya et al., 2021), so the secreted proteins in DEGs were analyzed. In order to understand the overall distribution of DEGs, volcano maps of DEGs were constructed (Supplementary Figure S4A). The cluster diagram of sample to sample distance matrix was prepared for DEGs to calculate the direct correlation of samples (Supplementary Figure S4B). From the comparison made between M1 and WT (M1-*vs*-WT), a total number of 168 DEGs were obtained, 74 of which were up-regulated and 94 of which were down-regulated. From the comparison made between M2 and WT (M2-*vs*-WT), 76 DEGs were obtained, 45 of which were up-regulated and 31 of which were down-regulated. From the comparison made between M1 and M2 (M1-*vs*-M2), 4 DEGs were obtained, 2 of which were up-regulated and 2 of which were down-regulated (Figure 5A). Similar DEGs found among the comparisons were shown in a Venn diagram (Figure 5B). Finally, a total of 216 genes were identified as differentially expressed. To understand the roles of various biological processes and pathways throughout the infection process, we analyzed Gene Ontology (GO) and Kyoto Encyclopedia of Genes and Genomes (KEGG) enrichment of the 216 DEGs. GO analysis was performed to classify the 216 DEGs into 3 major biological categories. The 216 DEGs belonged to 18 classes in the biological process category, 11 classes in the cellular component category, and 7 classes in the molecular function category (Figure 5C). In total, 15 secreted proteins were identified among the 216 proteins encoded by the DEGs using SignalP 5.0 (Table 2). 4 proteins among 15 proteins were predicated to be secreted proteins, PTTG_25982, PTTG_25534, PTTG_04712 and PTTG_04779 were identified as cysteine-rich proteins with the percentage of cysteine greater than or equal to 3% (Table 3).

**Table 2:** Characterization of 15 secreted proteins of *Pt.*

Gene name	Protein ID	Start	End	AA	SPL (%)	Description
*PTTG_25982*	OAV97527.1	283,774	284,535	177	99.67	—
*PTTG_00528*	OAV99683.1	1,293,484	1,294,592	245	98.61	—
*PTTG_05706*	OAV91377.1	99,128	100,145	180	98.49	—
*PTTG_08504*	OAV96694.1	959,195	960,977	261	98.5	—
*PTTG_11983*	OAV96685.1	846,080	847,237	200	97.66	—
*PTTG_25529*	OAV98838.1	843,242	844,456	165	99.87	—
*PTTG_25534*	OAV98849.1	938,968	940,077	202	98.74	—
*PTTG_02944*	OAV96672.1	803,183	804,937	242	99.8	—
*PTTG_02997*	OAV95795.1	123,092	124,677	188	98.95	Chitin deacetylase ARB_04768
*PTTG_04712*	OAV91195.1	364,128	365,858	217	99.65	—
*PTTG_26199*	OAV96956.1	706,361	707,112	181	99.35	—
*PTTG_26534*	OAV95830.1	464,431	465,799	173	97.11	—
*PTTG_04779*	OAV95943.1	830,568	832,307	252	99.08	Thaumatin-like
*PTTG_07625*	OAV97261.1	671,834	672,602	87	99.3	—
*PTTG_08990*	OAV98722.1	1,550,599	1,552,165	200	99.5	—

SPL, signal peptide likelihood.

**Table 3:** Characterization of 4 secreted proteins, which identified as cysteine-rich proteins.

Gene name	FPKM-WT	FPKM-M1	FPKM-M2	Foldchange (M1/WT)	Foldchange (M2/WT)	AA	Cys
*PTTG_25982*	91.15	544.89	–	5.97	–	177	11
*PTTG_25529*	158.62	337.91	–	2.00	–	165	12
*PTTG_04712*	47.36	128.06	365,858	2.72	–	217	8
*PTTG_04779*	2565.18	–	5154.76	–	2.00	252	16

AA, Amino acid; Cys, Cysteine residues.

### 3.7. PTTG_04779 inhibition the progress of cell death (PCD) induced by B-cell lymphoma-2 associated X protein (BAX) in *N. benthamiana*

**Figure 6:**
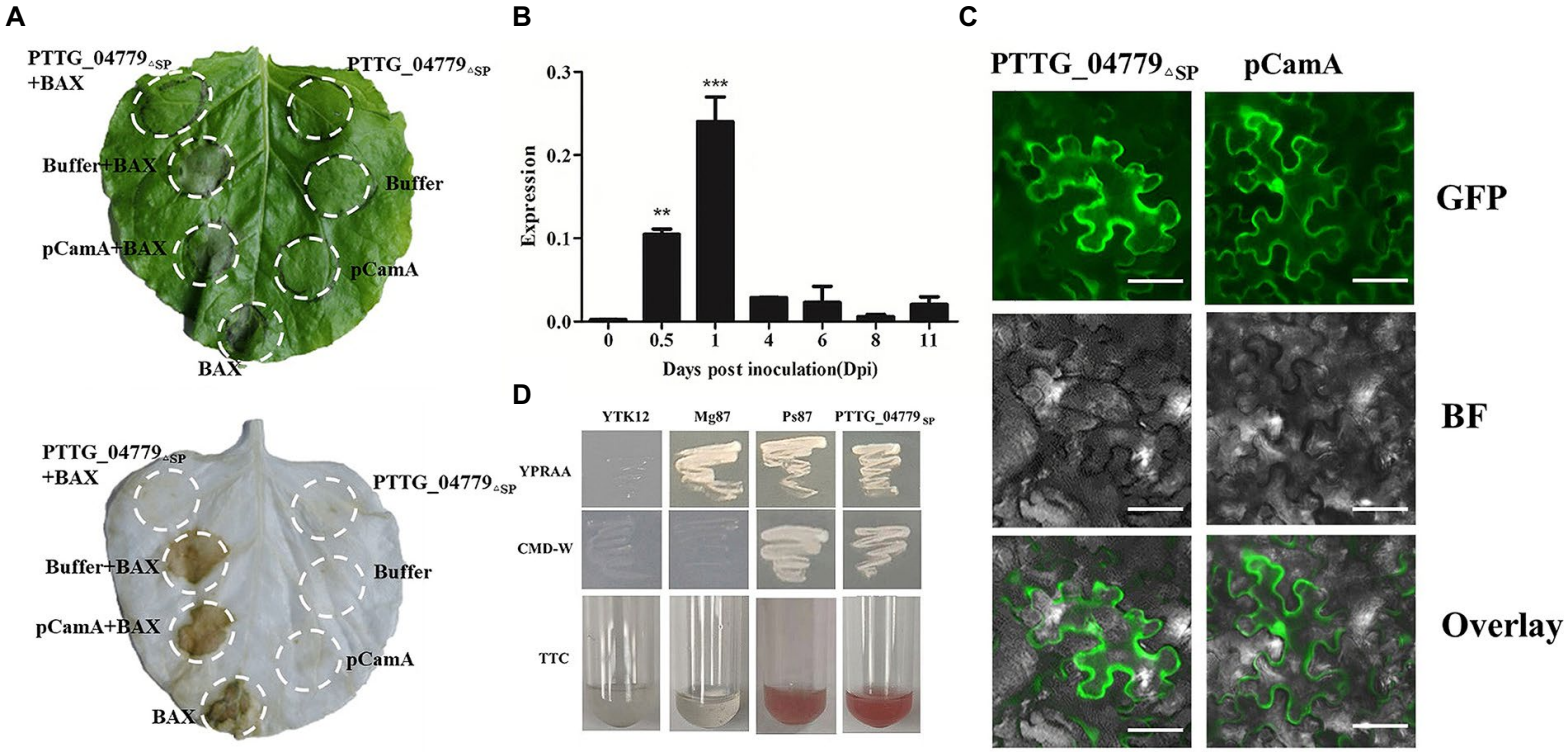
Characterization of secreted protein PTTG_04779. **(A)** PTTG_04779 can suppress BAX-induced cell death. PTTG_04779 was infiltrated into *N. benthamiana* leaves, followed 24 h later by infiltration with *A. tumefaciens* carrying the Bax gene. pCamA and buffer served as a negative. **(B)** Transcriptional profile of genes during the *Pt* pathotypes infection measured by qPCR. **(C)** Expressed fluorescently tagged PTTG_04779_ΔSP_ location in the *N. benthamiana* leaves epidermal cells. The pCamA-PTTG_04779_ΔSP_ (with GFP tag) in N. benthamiana cells and GFP field show green. Scale bar, 50 μm. **(D)** Yeast secretion trap assay of the secreted protein PTTG_04779. The predicted signal peptide coding sequences of PTTG_04779 (PTTG_04779_SP_) were cloned into the yeast secretion trap vector pSUC2. The Ps87 was used as a positive control, empty vector and Mg87 was used as a negative control.

Numerous avirulence proteins such as AvrM and AvrL567 from *Melampsora lini* (*Ml*) have been confirmed to be related to virulence of the pathogen (Ellis et al., 2007). To examine the function of secreted proteins, we test whether the secreted proteins could inhibit PCD. After transiently expressed the coding regions of 4 secreted proteins in *N. benthamiana* using *Agrobacterium* infiltration, the results showed that leaf regions co-expressing pCamA as a negative control and BAX developed PCD (Figure 6A), PTTG_25982, PTTG_25534 and PTTG_04712 could not suppressed PCD triggered by the expression of *BAX* gene (GenBank accession NP_031553) in *N. benthamiana* leaves (Supplementary Figure S5). However, there was no necrosis at the sites where BAX and PTTG_04779 were infiltrated together (Figure 6A), indicating that PTTG_04779 inhibited BAX function, preventing PCD from being induced, with potential toxic function.

qPCR was used to detect the expression levels of *PTTG_04779* at different time after *Pt* infection, and the results displayed that *PTTG_04779* expression peaked at 1 dpi which was 118-fold higher than that at 0 dpi (Figure 6B). Subcellular localization study showed that PTTG_04779_△SP_ (without signal peptide) located in the cytoplasm and nuclear of *N. benthamiana* cell (Figure 6C). To examine the function of the predicted signal peptide of PTTG_04779, a yeast signal sequence trap system was employed. Consistent with the positive control Ps87, YTK12 strains carrying the predicted signal-peptide coding sequences of PTTG_04779 (PTTG_04779_SP_) fused to pSUC2 were able to grow on YPRAA plates (Figure 6D). As expected, they were also able to catalyze the conversion of colorless 2,3,5-triphenyltetrazolium chloride (TTC) to red-colored triphenylformazan (TFP) (Figure 6D). Conversely, the negative control (YTK12 with empty vector) and Mg87 was not able to grow on a YPRAA plate and did not induce a TTC to TFP color change (Figure 6D). These results demonstrated that the predicted signal peptides of PTTG_04779 are functional. All these results suggest that *PTTG_04779* should be further studied as *AvrLr19* candidate gene. Taken together with 7 genes in *Lr19*-virulent mutants whose expression were affected by EMS mutation, there are total 8 genes as *AvrLr19* candidate (Table 4).

**Table 4:** A list of candidate genes for being *AvrLr19.*

Gene_name	Chr	Gene_start	Gene_end	Gene_length	Association type	SignalP D score	Effector *p*
*PTTG_27471*	ADAS02000057.1	14,998	15,372	375	SNP	0.662	0.882
*PTTG_12441*	ADAS02000035.1	163,086	164,553	1,227	SNP	0.764	0.878
*PTTG_28324*	ADAS02000101.1	313,403	315,005	1,384	SNP	0.591	0.621
*PTTG_26499*	ADAS02000024.1	315,857	316,639	698	SNP	0.703	0.523
*PTTG_06910*	ADAS02000138.1	17,746	19,966	1,748	SNP	0.603	-
*PTTG_26516*	ADAS02000024.1	908,360	908,725	366	SNP	0.506	0.506
*PTTG_03570*	ADAS02000017.1	494,282	495,594	1,072	Indel	0.713	0.606
*PTTG_04779*	ADAS02000024.1	830,568	832,307	1,739	DEG	99.08	0.908

Chr, Chromosome. SignalP D scores > 0.45 were considered positive predictions. Effector *p* > 0.50 were considered positive predictions.

## 4. Discussion

*Pt* is the causative agent of leaf rust, the most widespread and devastating disease of wheat, which resulted in yield losses of more than 50% in a single crop season. The obligate biotrophy and the lack of a transformation system for *Pt* complicates validation of the function of *Avr* gene from *Pt* and their host targets. Thus, different approaches to address the questions of virulence and avirulence in *Pt* had to be used. EMS mutagenesis have proved to be a useful tool to provide these answers. Li et al. (2019) developed a *Puccinia striiformis* f. sp. *tritici* (*Pst*) mutant population through EMS mutagenesis and characterized the population with virulence and molecular markers. Salcedo et al. (2017) obtained EMS induced urediniospore mutants from the wheat stem rust pathogen *Puccinia graminis* f. sp. *tritici* (*Pgt*) Ug99, which led to the cloning of *Avr* gene *AvrSr35*. Mutagenesis integrated with genomic sequencing is an efficient way to study the relationships between phenotypic traits and associated genes, leading to the identification of fungal effectors or avirulence genes. The innovation of the present study is that we developed two *Lr19*-virulent mutants *via* EMS mutagenesis, M1 and M2, and confirmed that the mutation site only altered the virulence of *Pt* race PHNT to *Lr19*, but not other *Lr* genes, which benefits the screening of *AvrLr19* candidates.

With the rapid development of sequencing technologies, the genome sequences of pathogen are available, which makes it possible to further understand the pathogenesis of the obligate biotrophic fungal parasite (Steuernagel et al., 2016). McDonald and Linde (2002) identified five *Pst* candidate effector genes from 2,999 predicted secreted protein genes. Xia et al. (2017) predicted a set of 25 *Pst Avr* candidate genes from 2,146 predicted secreted protein by combining comparative genomics with association analyses. Similar approaches were also used in detecting *Avr* candidate genes in *Pt* (Wu et al., 2017). Mutations in the *AvrLr19* coding region may lead to amino acid changes and loss of protein interaction with *Lr19*. To determine and characterize the potential *Avr* genes in *Pt*, whole-genome sequences of EMS-induced mutants were generated and analyzed. The reads of each isolate were mapped directly against the *Pt* Race 1 reference genome for SNP detection in a similar way to several previous studies (Cantu et al., 2013). An average of 240, 945 SNPs and 22, 361 Indels per isolate was found. This rate of mutation site matches that reported for the *Pt* race 1 genome (Persoon et al., 2014). This feature has been consistently noted in the genomes of other rust fungi, such as *M. larici-populina*, *Pgt* and *Pst* (Upadhyaya et al., 2021).

It was reported that a frequency of 99% G/C to-A/T transitions observed in *Arabidopsis* (Persoon et al., 2014). Thus, C/G-to-T/A mutations from the variants were selected, the other types of mutations were filtered out in this study, which is the same strategy used on the mutation screening work on tomato (Zheng et al., 2013), and fungal pathogen *Pgt* (Salcedo et al., 2017). 560 common genes containing SNPs and Indels mutation with SignalP D scores higher than 0.50 were predicted as secreted proteins by SignalP 5.0 (Mori et al., 2015; Nielsen et al., 2019). Salcedo et al. (2017) identified a fungal secreted protein named *AvrSr35* that is required for *Sr35* avirulence by whole-genome sequencing of EMS mutagenized and natural *Pgt* isolates. *AvrSr35* had a nonsynonymous mutation producing valine to isoleucine (V128I) substitution in mutation. In this study, 7 genes, *PTTG_27471*, *PTTG_03570*, *PTTG_26282*, *PTTG_28324*, *PTTG_06910*, *PTTG_26516* and *PTTG_26499*, from 560 common genes occurred SNPs and Indels mutation resulting amino acids, and the expression levels of these genes were significantly higher in *Lr19*-virulent mutants than that in WT, which indicated that they might be involved in the infection of TcLr19 and should be further study as *AvrLr19* candidate genes. Domain analysis was performed using InterProScan (Supplementary Figures S6A–G) and HMMER (Supplementary Figures S7A–G), no conserved domains were found in these 7 genes. Lots of fungi protein without known domain can play a pathogenic or avirulent role (Salcedo et al., 2017; Qi et al., 2019) and affect the process of fungi infection. In this study, the function of the candidate genes without known conserved domain will be further verified in the follow-up study.

It is attainable that the use of expression profiling to prioritize candidate genes for avirulence function in rust fungi. 216 DEGs obtained in this study may play a certain role in infection of M1 and M2 on TcLr19, and 15 proteins were predicated to be secreted proteins from them. It has been reported that cysteine is important for the function of proteins (Huang et al., 2019; Zhang et al., 2021). Therefore, PTTG_25982, PTTG_25534, PTTG_04712 and PTTG_04779 which identified as cysteine-rich proteins were paid much attention in this study. Among them, PTTG_04779 can suppress PCD, and its expression level is peaked at the stage of leaf rust infection, indicating that it might play a virulence function in plant. Domain analysis showed that there is a conserved domain of thaumatin in PTTG_04779 (Supplementary Figures S6H, S7H). It has been reported that the thaumatin-like family can regulate reactive oxygen species (ROS) production in plants and affect the disease resistance response of plants (Cui et al., 2021). Whether PTTG_04779 may play a thaumatin-like function in leaf rust similar to plants need to be test in the future. Moreover, to identify whether total 8 candidate genes conform to the characteristics of the effector, Effector P 3.0 was used. The results showed that 7 candidates were identified as effectors except PTTG_06910 (Table 4). However, some atypical secretory proteins do not conform to the characteristics of effector, but can affect the immune response of plants as pathogenic factors (Liu et al., 2014), so the function of PTTG_06910 need to be test.

As we all know the genes with signal peptide have a higher priority functional verification in the transcriptome sequencing data, but the candidates without signal peptide should not been discarded, because we found that not all the identified avirulence genes encoding secreted proteins. For example, *Magnaporthe oryzae* (*M. oryzae*) *Avr* gene *ACE1* is predicted to be non-secreted and localizes in the cytoplasm of appressoria (Greene et al., 2003). Therefore, non-SP genes obtained from variants with high associations to avirulence will be evaluated in the future. By analyzing the transcriptomes of all samples, we found that the ratio of M1_sample 1, M1-sample 3, and M2-sample 6 to the reference genome were lower than other samples. It may be due to insufficient spores in the sample. It was reported that 15 *Sr35*-virulent mutants generated by EMS were used to clone *AvrSr35* (Salcedo et al., 2017). More *Lr19*-virulent mutants need to be obtained and sequenced in order to improve the power of this study. Moreover, tools such as the effector-to-host analyser *Pseudomonas fluorescens* (*EtHAn*) strain-mediated effector delivery system (a bacterial type III secretion system) provide useful approaches for functional characterization of rust fungal effectors (Upadhyaya et al., 2014). The function of *AvrLr19* candidates and pathogenesis-related genes of *Pt* will be identified by using the bacteria type III secretion system in the future. By defining *AvrLr19* and its key functions, *AvrLr19* can be used to develop gene-specific molecular markers for directly monitoring *Pt* population changes in the field, which can be used for real-time monitoring of leaf rust races combined with marker-assisted selection (MAS) of wheat leaf rust resistance genes to predict the resistance of varieties and inform the correct use of resistant varieties. Our results will provide valuable resources for identifying and characterizing *AvrLr19* candidates and pathogenic genes, and lay a foundation for further elucidating the pathogenic mechanism of *Pt* and analyzing the disease-resistance mechanism of *Lr19*.

## Data availability statement

The datasets presented in this study can be found in online repositories. The names of the repository/repositories and accession number(s) can be found in the article/Supplementary material.

## Author contributions

DD, DL, and HW conceived and coordinated the study, designed the experiments, provided materials and resources, interpreted data, and revising the manuscript. WW and ZC participated in developing and increasing urediniospores of the mutant isolates, data analyses, draft the manuscript, and bioinformatic analysis of RNAseq data. FW and ZC participated in selecting isolate, producing spores, inoculating plants, and virulence data analysis. All authors read and approved the final manuscript.

## Funding

This work was supported by the National Natural Science Foundation of China (32172384, 31501623), the Natural Science Foundation of Hebei (C2020204028). The funding bodies played no role in the design of the study and collection, analysis, and interpretation of data and in writing the manuscript. Authors declare that they have no known competing financial interests or personal relationships that could have appeared to influence the work reported in this article.

## Conflict of interest

The authors declare that the research was conducted in the absence of any commercial or financial relationships that could be construed as a potential conflict of interest.

## Publisher’s note

All claims expressed in this article are solely those of the authors and do not necessarily represent those of their affiliated organizations, or those of the publisher, the editors and the reviewers. Any product that may be evaluated in this article, or claim that may be made by its manufacturer, is not guaranteed or endorsed by the publisher.

## Footnotes

^1^ https://www.ebi.ac.uk/interpro/about/interproscan/

^2^ https://www.ebi.ac.uk/Tools/hmmer/

^3^ https://effectorp.csiro.au/
