# Supplementary material for: Transcriptome analysis of Lr19-virulent mutants provides clues for the AvrLr19 of Puccinia triticina
Source: Front Microbiol. 2023 Mar 22;14:1062548. doi: 10.3389/fmicb.2023.1062548 (PMC10073493; doi:10.3389/fmicb.2023.1062548)

**Supplementary Figure 2.** Analysis of RNA sequencing

(A) The result of RNA quality detection. (B) Gene expression density in each sample.


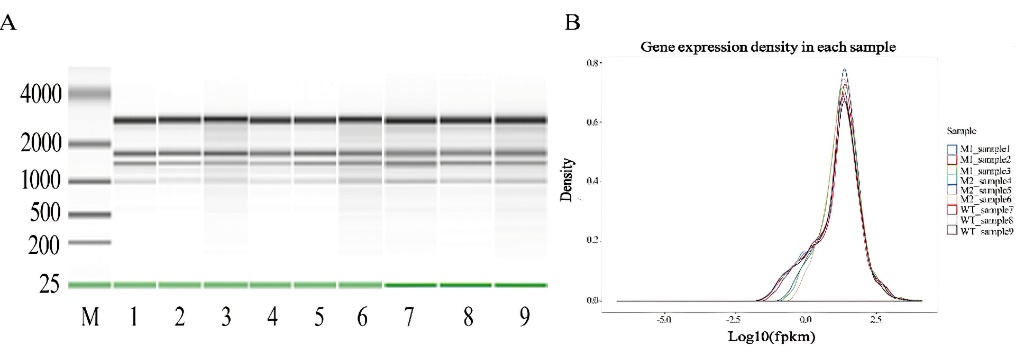


**Supplementary Figure 1.** Confocal microscopy of wheat leaves from the compatible Chinese Spring cultivar infected with the WT , M1 and M2 was performed to investigate the effect of the *AvrLr19* gene mutations on pathogen virulence. AP, appressorium; IH, infection hyphae; SH, secondly hyphae; Scale bars, 100 μm.


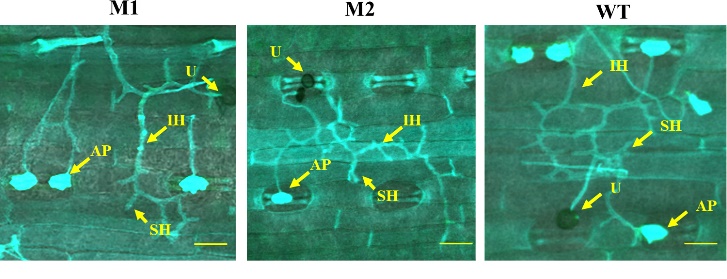


**Supplementary Figure 3.** Analysis of variance at different samples

(A) Box-whisker Plot; (B) Gene expression in each sample. (C) Principal component analysis (PCA) displays a clear distinction among the transcriptome of leaf rust at different samples**.**


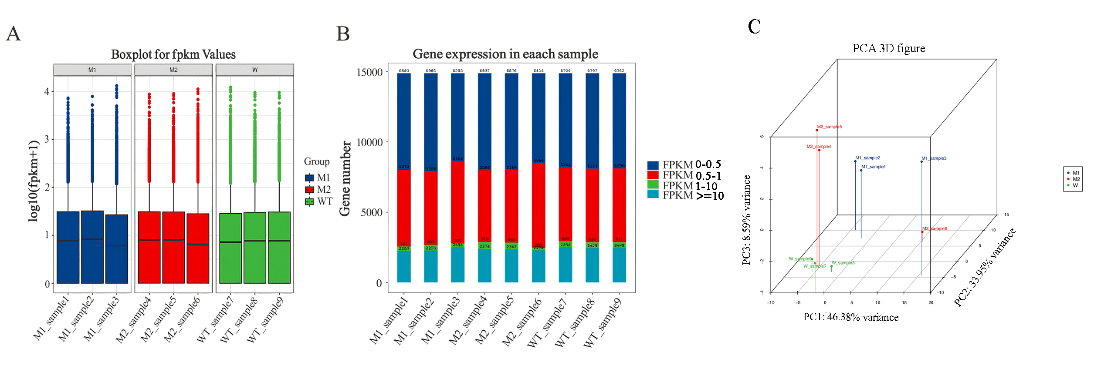


**Supplementary Figure 4.** The differentially expressed genes (DEGs)

(A) The volcano map, red dot indicates significantly up-regulated gene, green dot indicates significantly down regulated gene, gray dot indicates no significant difference gene; (B) Clustering of the sample-to-sample distances, red indicates significantly up-regulated genes and blue indicates significantly down regulated genes.


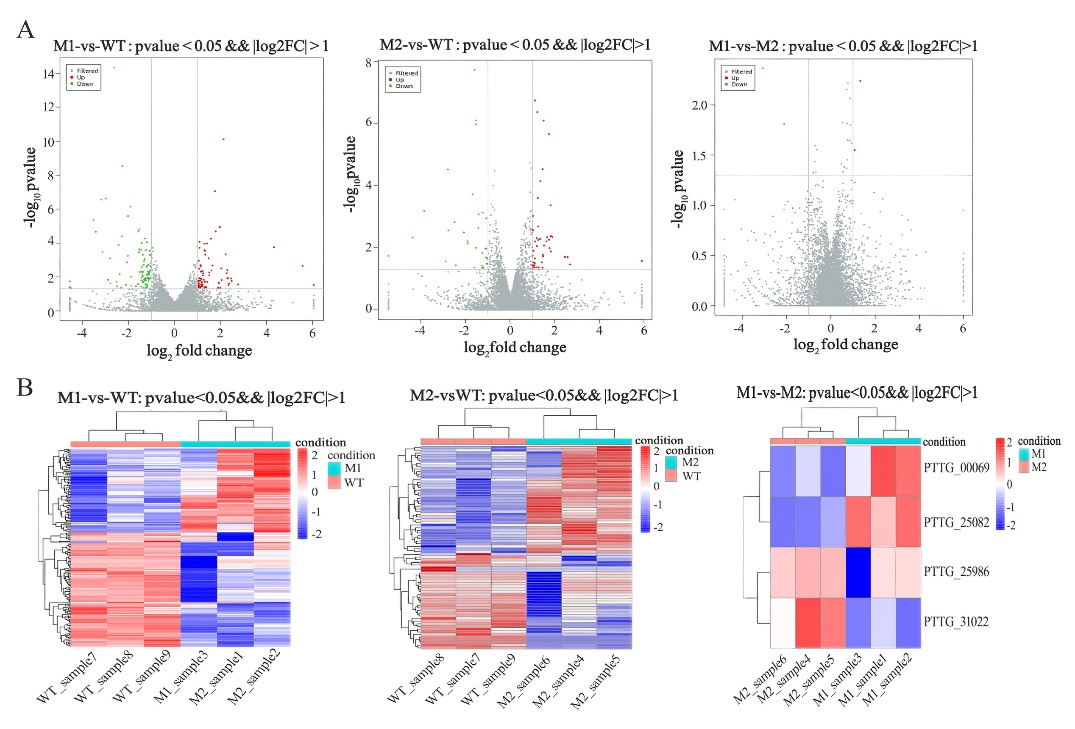

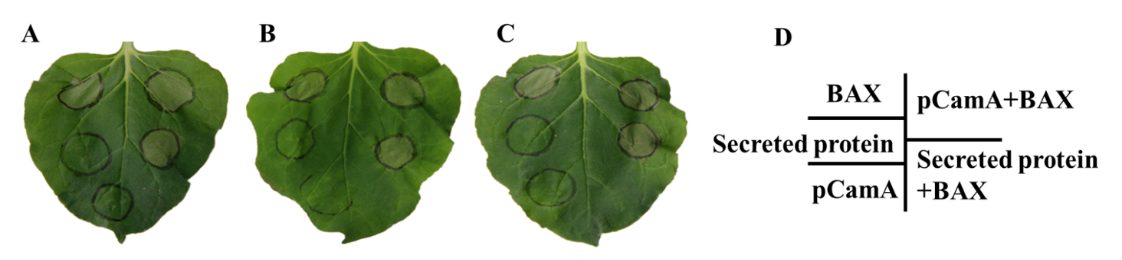


**Supplementary Figure 5.** 3 secreted proteins couldn’t inhibit programmed cell death (PCD) induced by BAX in *N. benthamiana.*

(A) PTTG_25534 , (B) PTTG_04712, (C) PTTG_25982 , (D) It’s the schematic of injection.

**Supplementary Figure 6. Analysis of conserved domains of *AvrLr19* candidate genes by InterProScan.** (A) PTTG_27471, (B) PTTG_28324, (C) PTTG_26499, (D) PTTG_06910, (E) PTTG_03570, (F) PTTG_26516, (G) PTTG_26282, (H) PTTG_04779.


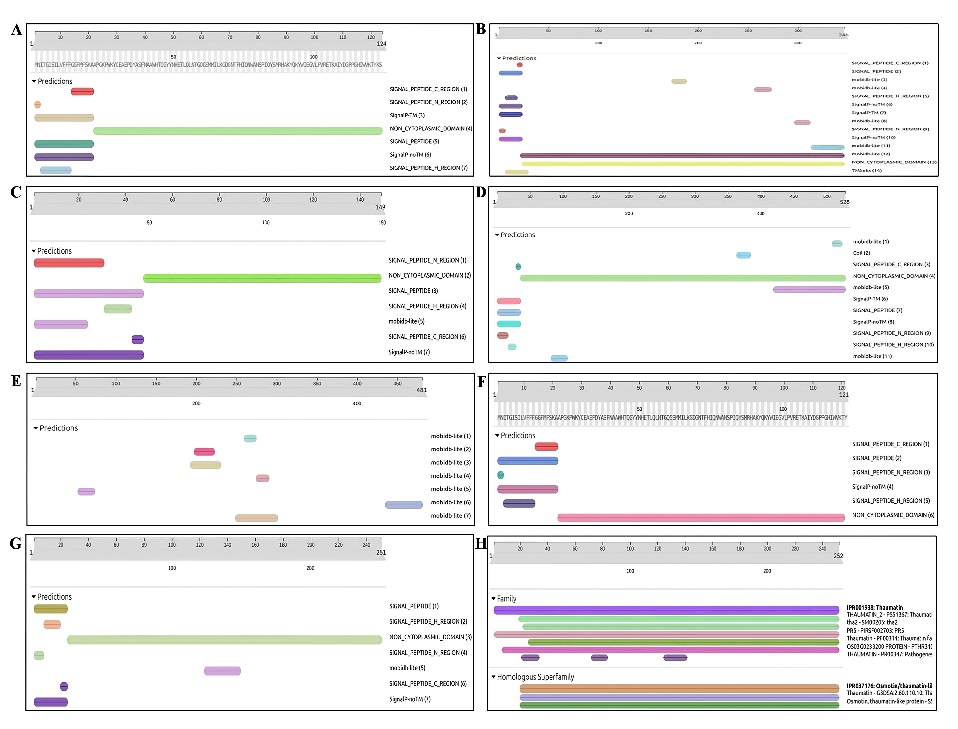


**Supplementary Figure 7. Analysis of conserved domains of *AvrLr19* candidate genes by HMMER.** (A) PTTG_27471, (B) PTTG_28324, (C) PTTG_26499, (D) PTTG_06910, (E) PTTG_03570, (F) PTTG_26516, (G) PTTG_26282, (H) PTTG_04779.


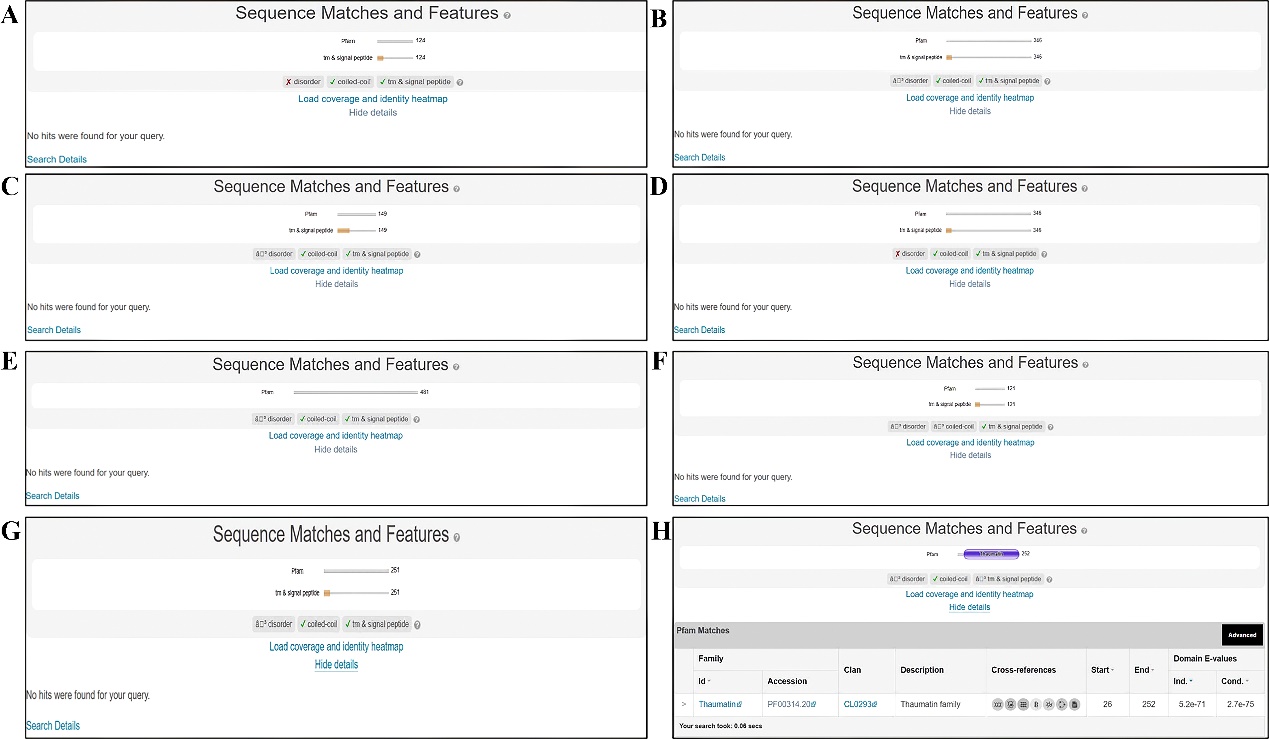

Supplement: Supplementary file 1 [file Data_Sheet_1.docx]
