# Supplementary material for: Transcriptome analysis of Lr19-virulent mutants provides clues for the AvrLr19 of Puccinia triticina
Source: Front Microbiol. 2023 Mar 22;14:1062548. doi: 10.3389/fmicb.2023.1062548 (PMC10073493; doi:10.3389/fmicb.2023.1062548)
Supplement: Supplementary file 2 [file Data_Sheet_2.docx]

**Supplementary Table 1** Infection types (IT)* on seedlings using the wild type *Pt* PHNT race and two EMS mutants of *Pt*, M1 and M2, against the differential set of wheat lines carrying different *Lr* genes.

| **ID** | **Wheat line** | ***Lr* gene** | **WT** | **M1** | **M2** |
| --- | --- | --- | --- | --- | --- |
| 1 | TcLr1-RL6003 | *Lr1* | 3 | 3 | 3 |
| 2 | TcLr2a-RL6016 | *Lr2a* | ;, 1 | ;, 1 | ;, 1 |
| 3 | TcLr2b-RL6019 | *Lr2b* | 4 | 4 | 4 |
| 4 | TcLr2c-RL6047 | *Lr2c* | 3+ | 3+ | 3+ |
| 5 | TcLr3-RL6002 | *Lr3* | 4 | 4 | 4 |
| 6 | TcLr3ka-RL6007 | *Lr3ka* | 3+ | 3+ | 3+ |
| 7 | TcLr3bg-RL6042 | *Lr3bg* | 3+ | 3+ | 3+ |
| 8 | TcLr9-RL6010 | *Lr9* | 0 | 0 | 0 |
| 9 | TcLr10-RL6004 | *Lr10* | 3+ | 3+ | 3+ |
| 10 | TcLr11-RL6053 | *Lr11* | ;, 1 | ;, 1 | ;, 1 |
| 11 | TcLr14a-RL6013 | *Lr14a* | 3 | 3 | 3 |
| 12 | TcLr14b-RL6006 | *Lr14b* | 3 | 3 | 3 |
| 13 | TcLr15-RL6052 | *Lr15* | 2 | 2 | 2 |
| 14 | TcLr16-RL6005 | *Lr16* | 3 | 3 | 3 |
| 15 | TcLr17-RL6008 | *Lr17* | 3+ | 3+ | 3+ |
| 16 | TcLr18-RL6009 | *Lr18* | 4 | 4 | 4 |
| 17 | TcLr19-RL6040 | *Lr19* | 0 | 3 | 3 |
| 18 | TcLr20-RL6092 | *Lr20* | ;, 1 | ;, 1 | ;, 1 |
| 19 | TcLr21-RL6043 | *Lr21* | 3 | 3 | 3 |
| 20 | TcLr23-RL6012 | *Lr23* | 2 | 2 | 2 |
| 21 | TcLr24-RL6064 | *Lr24* | 0, ; | 0, ; | 0, ; |
| 22 | TcLr25-RL6084 | *Lr25* | 3 | 3 | 3 |
| 23 | TcLr26-RL6078 | *Lr26* | 3+ | 3+ | 3+ |
| 24 | TcLr28-RL6079 | *Lr28* | 2+ | 2+ | 2+ |
| 25 | TcLr29-RL6080 | *Lr29* | ; | 0, ; | 0, ; |
| 26 | TcLr30-RL6049 | *Lr30* | ;, 1 | ;, 1 | ;, 1 |
| 27 | TcLr32-RL5497-1 | *Lr32* | 1+ | 1+ | 1+ |
| 28 | TcLr33-RL6057 | *Lr33* | 4 | 4 | 4 |
| 29 | TcLr36-E84018 | *Lr36* | 3 | 3 | 3 |
| 30 | TcLr38-RL6097 | *Lr38* | 0 | 0 | 0 |
| 31 | TcLr39-KS89WGRC02 | *Lr39* | 2+ | 2+ | 2+ |
| 32 | TcLr41-KS90WGRC10 | *Lr41* | 1 | 1 | 1 |
| 33 | TcLr44-RL6147 | *Lr44* | 3 | 3 | 3 |
| 34 | TcLr45-RL6144 | *Lr45* | 1 | 1 | 1 |
| 35 | Pavon76-Lr46 | *Lr46* | 0 | 0 | 0 |
| 36 | 90H450 | *Lr47* | 0, ; | 0, ; | 0, ; |
| 37 | Thatcher | *-* | 4 | 4 | 4 |

*Infection types (IT) at the seedling stage are based on Roelfs,where IT “ 0, ;, 1, 2,” or their combinations correspond to low infection types, IT “ 3 ” to “ 4 ” are considered high infection types. ITs are refined by adding -, when uredia are somewhat smaller than normal for the infection type, or +, when uredia are somewhat larger than normal for the infection type. Range of variation in ITs is shown by indicating the range with comma.

| **Sample** | **Raw_read** | **Raw_bases** | **Clean_reads** | **Clean_base** | **Valid_bases** | **Q30** | **GC** |
| --- | --- | --- | --- | --- | --- | --- | --- |
| M1_sample1 | 98.54M | 14.78G | 96.64M | 13.97G | 94.51% | 93.54% | 55.87% |
| M1_sample2 | 87.06M | 13.06G | 85.33M | 12.32G | 94.34% | 93.36% | 54.88% |
| M1_sample3 | 90.00M | 13.50G | 88.21M | 12.69G | 94.00% | 93.31% | 55.06% |
| M2_sample4 | 84.41M | 12.66G | 82.78M | 11.94G | 94.29% | 93.36% | 52.93% |
| M2_sample5 | 91.38M | 13.71G | 89.55M | 12.92G | 94.28% | 93.21% | 54.20% |
| M2_sample6 | 91.24M | 13.69G | 89.26M | 12.88G | 94.12% | 93.22% | 55.56% |
| W_sample7 | 98.79M | 14.82G | 96.63M | 13.93G | 94.00% | 93.11% | 52.47% |
| W_sample8 | 98.15M | 14.72G | 96.00M | 13.82G | 93.90% | 93.01% | 52.68% |
| W_sample9 | 90.13M | 13.52G | 88.24M | 12.77G | 94.46% | 93.14% | 52.99% |

**Supplementary Table 2** Sequencing data quality.

Sample: sample name;

Raw_reads: the number of original reads;

Raw_bases: the amount of original sequencing, i.e. basic orders;

Clean_reads: the number of clean reads after filtering;

Clean_bases: sequence quantity and base number after filtration;

Valid_base: effective base percentage;

Q30: the percentage of bases with Qphred value greater than 30 in raw bases;

GC: the percentage of the total number of G and C bases in clean bases.

| **Sample** | **Total reads** | | **Total mapped reads** | **Multiple mapped** | **Uniquely mapped** |
| --- | --- | --- | --- | --- | --- |
| M1_sample1 | | 96637830 | 5147403(5.33%) | 56476(0.06%) | 5090927(5.27%) |
| M1_sample2 | | 85331238 | 17198863(20.16%) | 217832(0.26%) | 16981031(19.90%) |
| M1_sample3 | | 88212260 | 3687907(4.18%) | 39567(0.04%) | 3648340(4.14%) |
| M2_sample4 | | 82778164 | 26735090(32.30%) | 336285(0.41%) | 26398805(31.89%) |
| M2_sample5 | | 89551190 | 24063082(26.87%) | 310127(0.35%) | 23752955(26.52%) |
| M2_sample6 | | 89255694 | 2257000(2.53%) | 24575(0.03%) | 2232425(2.50%) |
| W_sample7 | | 96634660 | 30570269(31.63%) | 433400(0.45%) | 30136869(31.19%) |
| W_sample8 | | 95998488 | 28464778(29.65%) | 387593(0.40%) | 28077185(29.25%) |
| W_sample9 | | 88236610 | 27026344(30.63%) | 355447(0.40%) | 26670897(30.23%) |
| Total reads: clean reads after sequencing data filtering;  Total mapped reads: Statistics of the number of sequencing sequences that can be located on the genome;  Multiple mapped: Statistics of the number of sequencing sequences with multiple alignment positions on the reference sequence;  Unique mapped: Statistics of the number of sequencing sequences with unique alignment positions on the reference sequence. | | | | | |

**Supplementary Table 3** The statistics of comparison rate between reads and reference genome

**Supplementary Table 4** Secreted protein genes with SNPs

| **Gene** | **Ref** | **Alt** | **FNC** | **Substitute** | **Chr.scaffold** |
| --- | --- | --- | --- | --- | --- |
| *PTTG_25160* | C | T | missense_variant | p.Ala160Thr/c.478G>A | ADAS02000001.1 |
| *PTTG_06256* | G | T | missense_variant | p.Gln482Lys/c.1444C>A | ADAS02000012.1 |
| *PTTG_26499* | G | A | missense_variant | p.His135Tyr/c.403C>T | ADAS02000024.1 |
| *PTTG_27005* | C | A | missense_variant | p.Thr89Asn/c.266C>A | ADAS02000040.1 |
| *PTTG_11739* | C | T | missense_variant | p.Arg135His/c.404G>A | ADAS02000042.1 |
| *PTTG_27471* | C | T | missense_variant | p.Gly70Glu/c.209G>A | ADAS02000057.1 |
| *PTTG_03597* | G | A | stop_gained | p.Gln119*/c.355C>T | ADAS02000071.1 |
| *PTTG_28324* | C | T | missense_variant | p.Ala167Thr/c.499G>A | ADAS02000101.1 |
| *PTTG_06910* | C | T | missense_variant | p.Ala377Thr/c.1129G>A | ADAS02000008.1 |
| *PTTG_00455* | G | T | missense_variant | p.Pro582Thr/c.1744C>A | ADAS02000128.1 |
| *PTTG_04011* | C | T | missense_variant | p.Pro458Leu/c.1373C>T | ADAS02000136.1 |
| *PTTG_26966* | C | T | missense_variant | p.Arg790Gln/c.2369G>A | ADAS02000108.1 |
| *PTTG_28035* | G | A | missense_variant | p.Ala622Val/c.1865C>T | ADAS02000104.1 |
| *PTTG_02175* | C | A | missense_variant | p.Leu20Met/c.58C>A | ADAS02000098.1 |
| *PTTG_05834* | C | A | missense_variant | p.Ala149Ser/c.445G>T | ADAS02000034.1 |
| *PTTG_28654* | G | T | missense_variant | p.Thr542Lys/c.1625C>A | ADAS02000035.1 |
| *PTTG_08941* | G | A | missense_variant | p.Gly142Ser/c.424G>A | ADAS02000038.1 |
| *PTTG_28961* | C | T | missense_variant | p.Pro85Ser/c.253C>T | ADAS02000118.1 |
| *PTTG_26282* | C | T | missense_variant | p.Ala206Thr/c.616G>A | ADAS02000018.1 |
| *PTTG_07374* | C | T | missense_variant | p.Gly215Glu/c.644G>A | ADAS02000001.1 |
| *PTTG_00363* | C | T | missense_variant | p.Arg725His/c.2174G>A | ADAS02000008.1 |
| *PTTG_07626* | G | A | missense_variant | p.Gly262Arg/c.784G>A | ADAS02000014.1 |
| *PTTG_26516* | G | A | missense_variant | p.Thr90Met/c.269C>T | ADAS02000024.1 |
| *PTTG_26586* | C | T | missense_variant | p.His130Tyr/c.388C>T | ADAS02000026.1 |
| *PTTG_11671* | G | T | missense_variant | p.Pro33Thr/c.97C>A | ADAS02000030.1 |
| *PTTG_12441* | C | T | missense_variant | p.Pro86Ser/c.256C>T | ADAS02000121.1 |
| *PTTG_28601* | G | A | missense_variant | p.Arg135Gln/c.404G>A | ADAS02000121.1 |
| *PTTG_30601* | G | A | missense_variant | p.Gly48Asp/c.143G>A | ADAS02004926.1 |
| *PTTG_06229* | C | T | missense_variant | p.Ala160Thr/c.478G>A | ADAS02000036.1 |
| *PTTG_02548* | C | T | missense_variant | p.Ala255Val/c.764C>T | ADAS02000161.1 |
| Ref: Reference bases on the genome.  Alt: Other base types detected.  FNC: Type of SNPs/ Indels location.  Substitute: Location of base substitutions and protein substitutions on genes.  Chr.scaffold: Chromosome. | | | | | |

**Supplementary Table 5** Secreted protein genes with Indels

| **Gene** | **Ref** | **Alt** | **FNC** | **substitute** | **chr.scaffold** |
| --- | --- | --- | --- | --- | --- |
| *PTTG_03570* | GTCCTC | GTCCTCCTCCTC | upstream_gene_variant | c.-116_-116insCTCCTC | ADAS02000017.1 |
| *PTTG_00981* | AAACCAGAATCAGAAC | AAACCAGAAT  CAGAACCAGA  ATCAGAAC | inframe_insertion | p.Gln60_Asn63dup/c.178_189dupCAGAATCAGAAC | ADAS02000019.1 |
| *PTTG_28601* | TC | T | upstream_gene_variant | c.-1delC | ADAS02000121.1 |
| *PTTG_29391* | TGG | TGGG | 5_prime_UTR_variant | c.-37_-36insC | ADAS02000358.1 |
| *PTTG_09239* | GGTGTG  TGTGT  GTGTGTGTGTGT | GGTGTGTGT  GTGTGTGTGTGT | 5_prime_UTR_variant | c.-421_-420delAC | ADAS02000567.1 |
| *PTTG_00016* | CTCCATCCATCCATCCATCCATCCATCCA | CTCCATCCATCCATCCATCCATCCA | 5_prime_UTR_variant | c.-40_-37delTCCA | ADAS02000004.1 |
| *PTTG_01827* | AAAAAGAAAA | AAAAA | 3_prime_UTR_variant | c.*121_*125delTTTTC | ADAS02000006.1 |
| *PTTG_03567* | TAGTCGGCCAG | TAG | 3_prime_UTR_variant | c.*437_*444delTCGGCCAG | ADAS02000020.1 |
| *PTTG_04011* | AG | A | 5_prime_UTR_variant | c.-20delG | ADAS02000029.1 |
| *PTTG_06895* | GC | G | frameshift_variant | p.Gln306fs/c.918delG | ADAS02000037.1 |
| *PTTG_27264* | TGTGAGTGAGTGAGTGAGTGAGTG | TGTGAGTGAGTGAGTGAGTG | upstream_gene_variant | c.-1_-1delCACT | ADAS02000049.1 |
| *PTTG_08198* | TTGATGTTTTTTTTGATGATG | TTGATG | downstream_gene_variant | c.*675_*675delTTTTTTTTGATGATG | ADAS02000180.1 |
| *PTTG_04053* | TGGCCG | TG | frameshift_variant | p.Ala200fs/c.599_602delCGGC | ADAS02000275.1 |
| *PTTG_02477* | AGGAATGGAGGGATCGAGGGAATGGAGGG | AGGAATGGAGGG | frameshift_variant | p.Pro696fs/c.2086_2102delCCCTCCATTCCCTCGAT | ADAS02000004.1 |
| *PTTG_28296* | AGG | AGGG | 3_prime_UTR_variant | c.*22_*23insC | ADAS02000100.1 |
| Ref: Reference bases on the genome.  Alt: Other base types detected.  FNC: Type of SNPs/ Indels location.  Substitute: Location of base substitutions and protein substitutions on genes.  Chr.scaffold: Chromosome. | | | | | |

**Supplementary Table 6.** List of primers used in the qPCR analysis

| **Primer names** | **Primer sequences (5'-3')** |
| --- | --- |
| PTTG_27471-F | AAGCAGAACCAGATTATG |
| PTTG_27471-R | CACCTTCAATCCAGTATT |
| PTTG_11671-F | ATGATGATGCTGAAC |
| PTTG_11671-R | CTGGTTCTTGTAGTC |
| PTTG_27005-F | AACGGCTTCAACTACTTC |
| PTTG_27005-R | CTTCTTGAACAGCACCTT |
| PTTG_28324-F | ACGCTGATAAGACAGACTC |
| PTTG_28324-R | GTTTCGCTTTGTGTGCTT |
| PTTG_26499-F | GTGTCTAACAGCTTCAAT |
| PTTG_26499-R | TTACGAGTCATTGGTAGG |
| PTTG_00069-F | CTGTTGAATCGCTCATTGA |
| PTTG_00069-R | ATTTGTCGCCACGTTTGA |
| PTTG_26562-F | TGTATATTCAGCCTTATG |
| PTTG_26562-R | TATTCTCATCTTCCATTC |
| PTTG_03570-F | TACTTCGGCAACGGCTAC |
| PTTG_03570-R | ATGTAGTTGAAGCACTGGTAG |
| PTTG_26282-F | GTACCTGTTCCTGTG |
| PTTG_26282-R | TCTTCTTGTGCTTCA |
| PTTG_12441-F | GGTGAACGATAAAGTAAT |
| PTTG_12441-R | CGATTCTATTGTCAACAT |
| PTTG_26516-F | ATGAAGATTACTGGAACA |
| PTTG_26516-R | GATGATGGTGATGCTATA |
| PTTG_06910-F | ACACTTTGTAGTTACCAT |
| PTTG_06910-R | AGTTCTTCCTTGAGATAG |
| PTTG_02413-F | CTATTCTCATACGGATAA |
| PTTG_02413-R | GAGGACCTATCAATAATC |
| PTTG_29962-F | ATATGAATGTCGCAGGAA |
| PTTG_29962-R | ACCTTCGTGTATGTAGTC |
| PTTG_04779_SP_-F | GAATTCATGAAGCAAGCTTTTGTG |
| PTTG_04779_SP_-R | CTCGAGTGCGGCGATACTGGAAGC |
| PTTG_04779_△SP_-F | GGATCCAACTGCCCTTTCACCA |
| PTTG_04779_△SP_-R  β-actin-F  β-actin-R | CCCGGGTTAGGGGCAGAATGTGA  GTTCTACAACGAGCTCCGTGTC  GACATACATTGCTGGGCAAC |
